# Interleukin-15 Signaling in HIF-1α Regulation in Natural Killer Cells, Insights Through Mathematical Models

**DOI:** 10.3389/fimmu.2019.02401

**Published:** 2019-10-16

**Authors:** Anna Coulibaly, Anja Bettendorf, Ekaterina Kostina, Ana Sofia Figueiredo, Sonia Y. Velásquez, Hans-Georg Bock, Manfred Thiel, Holger A. Lindner, Maria Vittoria Barbarossa

**Affiliations:** ^1^Department of Anesthesiology and Surgical Intensive Care Medicine, Medical Faculty Mannheim, University Medical Center Mannheim, Heidelberg University, Mannheim, Germany; ^2^Interdisciplinary Center for Scientific Computing, Heidelberg University, Heidelberg, Germany; ^3^Institute for Applied Mathematics, Heidelberg University, Heidelberg, Germany

**Keywords:** HIF-1α, IL-15, STAT3, NF-κB, mTOR, natural killer cells, parameter estimation, mathematical model

## Abstract

Natural killer (NK) cells belong to the first line of host defense against infection and cancer. Cytokines, including interleukin-15 (IL-15), critically regulate NK cell activity, resulting in recognition and direct killing of transformed and infected target cells. NK cells have to adapt and respond in inflamed and often hypoxic areas. Cellular stabilization and accumulation of the transcription factor hypoxia-inducible factor-1α (HIF-1α) is a key mechanism of the cellular hypoxia response. At the same time, HIF-1α plays a critical role in both innate and adaptive immunity. While the HIF-1α hydroxylation and degradation pathway has been recently described with the help of mathematical methods, less is known concerning the mechanistic mathematical description of processes regulating the levels of HIF-1α mRNA and protein. In this work we combine mathematical modeling with experimental laboratory analysis and examine the dynamic relationship between HIF-1α mRNA, HIF-1α protein, and IL-15-mediated upstream signaling events in NK cells from human blood. We propose a system of non-linear ordinary differential equations with positive and negative feedback loops for describing the complex interplay of HIF-1α regulators. The experimental design is optimized with the help of mathematical methods, and numerical optimization techniques yield reliable parameter estimates. The mathematical model allows for the investigation and prediction of HIF-1α stabilization under different inflammatory conditions and provides a better understanding of mechanisms mediating cellular enrichment of HIF-1α. Thanks to the combination of *in vitro* experimental data and *in silico* predictions we identified the mammalian target of rapamycin (mTOR), the nuclear factor-κB (NF-κB), and the signal transducer and activator of transcription 3 (STAT3) as central regulators of HIF-1α accumulation. We hypothesize that the regulatory pathway proposed here for NK cells can be extended to other types of immune cells. Understanding the molecular mechanisms involved in the dynamic regulation of the HIF-1α pathway in immune cells is of central importance to the immune cell function and could be a promising strategy in the design of treatments for human inflammatory diseases and cancer.

## 1. Introduction

As effector lymphocytes of innate immunity, natural killer (NK) cells are involved in the host defense against microbial infections and cancer (1). Sensing their environment, NK cells respond to cellular alterations including those caused by infections, cellular stress, and transformation (2).

Interleukin-15 (IL-15), produced by monocytes, macrophages and dendritic cells, critically regulates NK cell survival and activation (3, 4). While expression of IL-15 is low under homeostatic conditions, it is upregulated in inflammation (5). Upon receptor binding, IL-15 initiates Janus kinase/signal transducer and activator of transcription (JAK/STAT) signaling. This promotes growth of NK cells and enhances their ability to respond to activation. Activated NK cells infiltrate tissues containing pathogen-infected or malignant cells, resulting in their recognition and direct killing (6–8).

Sites of infection or cellular transformations are often characterized by inflammatory hypoxia. Thus, NK cells must adapt and respond under conditions of low oxygen tension. The critical cellular dependence of survival on oxygen led to the early evolution of adaptive cellular responses to hypoxia. Cellular adaptation to hypoxia is primarily orchestrated by the hypoxia inducible factor (HIF) family of transcription factors (9). To date, three HIF family members have been identified (HIF-1, HIF-2, and HIF-3) of which HIF-1 is the best characterized (10). Two subunits, HIF-1α and HIF-1β, form the transcriptionally active HIF-1 complex. The α-subunit is post-translationally hydroxylated by oxygen-sensitive prolyl hydroxylases (PHDs) which mark the protein for ubiquitination and continuous proteasomal degradation. A decrease in cellular oxygen availability stabilizes HIF-1α allowing its dimerization with HIF-1β. The dimer translocates to the nucleus, binds to hypoxia-response elements in promoters of adaptive genes, and activates their expression.

In immune cells, including T lymphocytes and myeloid cells, cellular activation of HIF-1α has also been reported to occur in an oxygen-independent manner during inflammation triggered by infection and cancer, and to involve transcriptional in addition to post-translational mechanisms (11). At sites of tissue damage and infection, both inflammation and decreased oxygen availability result in HIF-1α stabilization and its nuclear translocation. Recent insights from models of solid tumors in mice with an NK cell specific knockout of the HIF-1α gene and from chemical inhibition of HIF-1α in human NK cells (12, 13) suggest that HIF-1α limits NK cell anti-tumor activity.

In the past 15 years, several mathematical models for HIF-1α regulation based on systems of ordinary differential equations (ODEs) have been proposed (14–19). A review up to 2013 is given in (20). Nguyen and coauthors (19) have investigated the dynamics of the HIF-1α pathway, combining a mathematical mechanistic model and experimental analysis for human embryonic kidney 293 (HEK-293) cells. Their model studies accumulation of HIF-1α in hypoxia and its degradation in normoxia, considering hydroxylation of HIF-1α mediated through both prolyl hydroxylases and asparaginyl hydroxylase FIH (factor inhibiting HIF). Fábián et al. (10) have highlighted the importance of using system biology and mathematical modeling for understanding HIF signaling. Although lacking the comparison with experimental data, Fábián's models allowed to test different hypotheses on the HIF network, concluding that the negative feedback induced by PHDs plays a major role in triggering oscillations in the HIF-1α dynamics.

This work combines mathematical modeling and experimental analysis to understand processes regulating the levels of HIF-1α mRNA and protein in NK cells. The proposed mathematical model considers key features of HIF-1α regulation and is formulated as a system of non-linear ODEs with positive and negative feedbacks. In our *in vitro* studies, we isolated human peripheral NK cells and studied their behavior simulating hypoxic and inflammatory conditions, which were produced by the hypoxia-mimicking agent dimethyl-oxalyl glycine (DMOG) and the pro-inflammatory cytokine IL-15, respectively. Experimental trials were designed to collect time series data of HIF-1α protein expression and its upstream regulators in order to calibrate the mathematical model. Parameter estimation was performed by means of numerical methods based on a multiple shooting approach for dynamic systems and a generalized Gauss-Newton method for optimization. Our approach does not only explain experimental observations on HIF-1α dynamics but also allows to ask questions and test hypotheses with the help of *in silico* experiments. For example, we investigated how HIF-1α levels depend on the regulation of other upstream proteins, and identified the signal transducer and activator of transcription 3 (STAT3), the mammalian target of rapamycin (mTOR) and the nuclear factor-κB (NF-κB) as critical regulators. Further, we studied HIF-1α stabilization in dependence of DMOG-mediated PHD/FIH inhibition, determining a non-linear relation between HIF-1α levels and DMOG concentration. Our model provides new insights into the mechanisms mediating accumulation of HIF-1α in NK cells, by (i) highlighting the synergistic effects of IL-15 and chemical hypoxia, and (ii) suggesting that NF-κB and STAT3 are fundamental regulators of IL-15 induced HIF-1α enrichment.

## 2. Materials and Methods

### 2.1. NK Cell Purification and Cell Culture

The study was reviewed and approved by the Medical Ethics Commission II of the Medical Faculty Mannheim, Heidelberg University (2014-500N-MA). NK cells were isolated from buffy coats obtained through the local Red Cross Blood Donor Service (NK-Cell Isolation Kit, Miltenyi Biotec GmbH, Bergisch Gladbach, Germany). The purity of NK cells was determined by flow cytometry.

Freshly isolated NK cell preparations with a phenotype of ≥95% CD56^+^CD3^−^ and ≤1% each CD3^+^, CD14^+^, CD15^+^, and CD19^+^ were judged as pure and were further cultivated as previously described (21). In brief, cells were plated at a density of 10^6^ cells/mL in RPMI 1640 medium (Sigma-Aldrich Chemie GmbH, Merck KGaA, Darmstadt, Germany) supplemented with 10% fetal bovine serum (FBS) and 2 mM L-glutamine and maintained in a standard tissue culture incubator (37°C, 5% CO_2_, 21% O_2_, normoxia, standard condition). The cell permeable pan-hydroxylase inhibitor DMOG (Selleck Chemicals, Houston, TX, USA) was used to mimic hypoxia. The viability of the cells was determined by tryptan blue staining and was ≥95% (Countess, Invitrogen, ThermoFisher, Waltham, MA, USA).

### 2.2. *In vitro* Treatments

Freshly isolated NK cells were maintained overnight under standard conditions and were stimulated with human recombinant IL-15 (45 ng/mL, PeproTech, NJ, USA), DMOG (20 μM, Selleck Chemicals), rapamycin (25 nM, Merck Chemicals GmbH, Darmstadt, Germany), STAT3 inhibitor (S3I-201, 200 μM, Merck Chemicals GmbH), or DMSO (Sigma-Aldrich Chemie GmbH) as control, on the next day for the indicated time periods. Protein concentrations in cell lysates were determined on a Direct Detect® infrared spectrometer (Merck Millipore) according to the manufacturer's instructions.

### 2.3. Western Blotting

Total cell extracts were prepared by resuspending 3 × 10^6^ NK cells in 100 μL NP-40 lysis buffer (50 mM Tris-HCl, pH 7.5, 120 mM NaCl, 20 mM NaF, 1 mM EDTA, 6 mM EGTA, 15 mM sodium pyrophosphate, 1 mM PMSF, 0.1% Nonident P-40). Fifteen minutes of cell lysis on ice was followed by centrifugation for 20 min at 14,000 × g. Cleared lysates were analyzed directly by SDS-PAGE and Western Blotting. Briefly, equal amounts of protein were separated by SDS-PAGE, transferred to nitrocellulose membranes (Thermo Fisher), blocked in 5% dry milk powder dissolved in 1×PBS-T, and then probed with primary antibody and HRP-conjugated secondary antibody (Santa Cruz Biotechnology, Dallas, TX, USA). Proteins were visualized using Enhanced Chemiluminescent solution (Thermo Fisher) and FUSION Vilber imager (Eberhardzell, Germany). The intensity of signals was quantified by densitometric analysis using the image analysis software ImageJ (Version 1.51j8). The value for HIF-1α was normalized to that for β-Actin. Anti-HIF-1α (# 2185) was obtained from Abcam (Cambridge, UK) and Anti-Actin (8H10D10) from Cell Signaling Technology (Frankfurt am Main, Germany). Representative experiments out of three performed are shown.

### 2.4. MILLIPLEX Immunoassay

The MILLIPLEX MAP Multi-Pathway Signaling Phosphoprotein Kit 48-680MAG was used according to the manufacturer's protocol (Merck Millipore). Total NK cell extracts were diluted with MILLIPLEX MAP Assay Buffer to reach the protein concentration of 10 μg of total protein/well. Mixed magnetic beads were added to each well. To appropriate wells, 25 μL of Assay Buffer (background control), 25 μL of NK cell sample lysates and 25 μL of control cell lysates were added in duplicates. The plate was sealed and incubated overnight (20 h) at 4°C on a plate shaker (750 rpm). After incubation and washing, 25 μL of Detection Antibody were added to each well. This incubation step was followed by addition of 25 μL Streptavidin-Phycoerythrin and incubation on the shaker. After resuspending the beads in 150 μL of Assay Buffer, the plate was read on a MAGPIX system (Luminex). Signals of phosphorylation of STAT3 and AKT were expressed as background-corrected median fluorescence intensities.

### 2.5. Modeling the Regulatory Network

**Figure 1:**
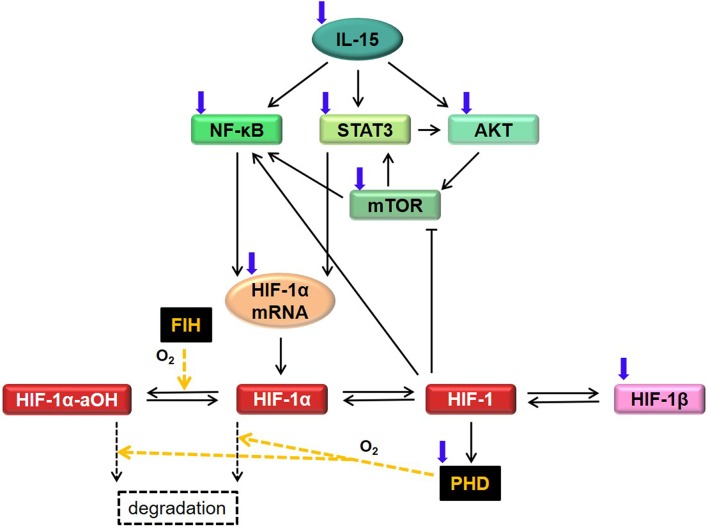
Diagram for HIF-1α regulatory network in NK cells, corresponding to model Equations (2)–(11). We study the interplay of HIF-1α, IL-15, mTOR, NF-κB, and STAT3 in normoxia and hypoxia. A signaling cascade starting with IL-15 activates NF-κB, STAT3, and AKT. This in turn activates mTOR, influencing HIF-1α mRNA and HIF-1α protein levels. In normoxia, HIF-1α is hydroxylated via FIH and PHD. Here, we consider only the FIH-mediated asparaginyl-hydroxylated HIF-1α (HIF-1α-aOH) and assume that a small fraction of HIF-1α-aOH can be dehydroxylated. In normoxia, hydroxylated HIF-1α is degraded via Von Hippel-Landau protein (not considered in the model). In hypoxia, HIF-1α accumulates and binds to HIF-1β, building the HIF-1 complex. The latter is responsible for both a positive and a negative feedback on HIF-1α, via activation of NF-κB and via inhibition of mTOR and upregulation of PHD. Black arrows indicate protein activation, translation or formation/dissociation of protein complexes. For simplicity, basal degradation of molecules is not depicted here. The blind-ended arrow indicates inhibition. Blue arrows indicate external regulation due to further stimuli not specifically considered in the mathematical model. Yellow arrows indicate HIF-1α hydroxylation via FIH or PHD.

The mathematical approach used in this study is based on a system of autonomous non-linear ODE, which can be in general written as

(1) $$\begin{array}{l} \left\{ \begin{array}{ccc} y^{\prime }\left(t\right) & =f\left(y,u,p\right), & t\in \left[t_{0},t_{f}\right]\subset \mathbb{R}\\ y\left(t_{0}\right) & =y_{0}\left(p\right).\; & \; \end{array}\right. \end{array}$$

with states *y*, controls *u* and parameters *p*. The vector $y\left(t\right)\in {\mathbb{R}}^{n_{y}}$ indicates the “state of the system,” that is, the concentration of the considered proteins, complexes and mRNA at time *t* ∈ [*t*_0_, *t*_*f*_] ⊂ ℝ, and $y_{0}\in {\mathbb{R}}^{n_{y}}$ is the initial state. The parameter vector $p\in {\mathbb{R}}^{n_{p}}$ contains non-negative constants describing the biochemical reaction rates (such as production, degradation, binding, etc.) in the system. The time-dependent experimental controls $u\left(t\right)\in {\mathbb{R}}^{n_{u}}$ represent cell treatments, specifically with IL-15, DMOG, or other protein inhibitors. In this work we do not discuss basic theoretical properties of the solutions to system 1, such as existence and uniqueness of a global solution, or invariance of the positive cone of ${\mathbb{R}}^{n_{y}}$. All these properties can be proven by applying elementary results and methods in ODE theory [see e.g., (22)], and we assume them to hold true in this manuscript. In the following we explain in detail the model assumptions and the resulting equations. Model variables and parameters are given in Table S1, and Tables 1, 2, respectively. A diagram of the regulatory network is shown in Figure 1.

**Table 1:** Parameter description and values used for the mathematical model (2)–(11).

**Parameter (fixed)**	**Description [Unit]**	**Remark**
*a*_1_	IL-15 external regulation rate [nM h^−1^]	0 (steady state cond.)
*a*_2_ = 0.848	AKT basal activation rate [nM h^−1^]	Prefit + sens. anal.
*a*_3_ = 0.037	mTOR basal activation rate [nM h^−1^]	Prefit + sens. anal.
*a*_7_ = 0	NF-κB basal activation rate [nM h^−1^]	Biol. assumption
*a*_8_ = 0	STAT3 basal activation rate [nM h^−1^]	Biol. assumption
*a*_9_ = 0	HIF-1α mRNA basal synthesis rate [nM h^−1^]	Biol. assumption
*a*_11_ = 4.17	PHD equilibrium level in normoxia [nM]	(10, 19)
*d*_8_	STAT3 basal decay rate [h^−1^]	=*k*_8_ (steady state cond.)
ρ_6_ = 99%	Efficacy (0-100%) of DMOG	
	as PHD/FIH inhibitor [dim-less]	(19)
*K*_O_2__ = 0.96	FIH/PHD oxygen-dependent binding force	
	in normoxia [dim-less]	(19)
*k*_1_ = 2 · 10^−5^	AKT activation rate via IL-15 [h^−1^]	Prefit + sens. anal.
*k*_2_ = 0.307	mTOR activation rate via AKT [h^−1^]	Prefit + sens. anal.
*k*_5_ = 75.895	HIF-1 complex dissociation rate [h^−1^]	Prefit + sens. anal.
*k*_10_ = 421.353	Catalytic constant rate	
	for PHD mediated HIF-1α hydroxylation [h^−1^]	Prefit + sens. anal., cf. (19)
*k*_11_ = 0.211	Catalytic constant rate	
	for dehydroxylation of HIF-1α-aOH [nM h^−1^]	Prefit + sens. anal.
*k*_12_ = 0.061	Catalytic constant rate	
	for PHD mediated HIF1α-aOH hydroxylation [h^−1^]	Prefit + sens. anal.
*k*_15_ = 0.088	mTOR-induced NF-κB activation rate [h^−1^]	Prefit + sens. anal.
*k*_*S*_ = 9 · 10^−4^	Maximal STAT3-regulated	
	AKT activation rate [nM h^−1^]	Prefit + sens. anal.
*n*_2_ = 2	Hill coefficient in	
	STAT3-mediated AKT regulation [dim-less]	Biol. assumption, cf. (23)
ξ_28_ = 38.44	Threshold in STAT3-mediated	
	AKT regulation [nM]	Prefit + sens. anal.
Δ = 200	Hypoxia-regulated PHD production	(10, 19)
	at equilibrium [dim-less]	
ξ_4_ = 15.018	Michaelis-Menten constant	
	for HIF-1α as substrate of FIH [nM]	Prefit + sens. anal., cf. (19)
ξ_44_ = 128.022	Michaelis-Menten constant	
	for HIF-1α as substrate of PHD [nM]	Prefit + sens. anal., cf. (19)

*In the left column we report the parameter value used for the numerical simulations. In the right column we indicate whether the parameter value has been fixed because of previous literature, or pre-fitted and then fixed because of sensitivity analysis results (cf. section 2.7)*.

**Table 2:** Parameter description and values used for the mathematical model (2)–(11).

**Parameter**	**Description [Unit]**	**Estimated value (s.d.)**
*a*_5_	HIF-1β basal synthesis rate [nM h^−1^]	0.211 (0.047)
*d*_1_	IL-15 basal decay rate [h^−1^]	0.062 (0.006)
*d*_2_	AKT basal decay rate [h^−1^]	0.848 (0.080)
*d*_3_	mTOR basal decay rate [h^−1^]	0.919 (0.010)
*d*_4_	HIF-1α basal decay rate [h^−1^]	0.623 (0.052), cf. (19)
*d*_5_	HIF-1β basal decay rate [h^−1^]	0.196 (0.062)
*d*_6_	HIF-1 basal decay rate [h^−1^]	0.301 (0.043), cf. (19)
*d*_7_	NF-κB basal decay rate [h^−1^]	0.914 (0.030)
*d*_9_	HIF-1α mRNA basal decay rate [h^−1^]	0.934 (0.042)
*d*_10_	HIF-1α-aOH degradation rate [h^−1^]	0.935 (0.037)
ρ_3_	Efficacy of S3I-201 [dim-less]	100% (2.7%)
ρ_4_	Inhibitory effect of DMOG on	
	IL-15 mediated STAT3 activation [dim-less]	0.863 (0.007)
*k*_3_	STAT3-regulated HIF-1α mRNA production rate [h^−1^]	0.181 (0.017)
*k*_4_	Association rate of HIF-1α and HIF-1β [nM^−1^h^−1^]	76.196 (4.986)
*k*_6_	STAT3 activation rate via IL-15 [h^−1^]	25.001 (1.412)
*k*_7_	IL-15-regulated NF-κB activation rate [h^−1^]	2.903 (0.358)
*k*_8_	STAT3 activation rate via mTOR [h^−1^]	0.577 (0.052)
*k*_9_	NF-κB-regulated HIF-1α mRNA production rate [h^−1^]	0.753 (0.205)
*k*_13_	Catalytic constant rate	
	for PHD mediated HIF-1α hydroxylation [h^−1^]	12.152 (2.272)
*k*_14_	Hypoxia-induced NF-κB activation rate [h^−1^]	16.528 (3.824)
*k*_α_	HIF-1α synthesis rate [h^−1^]	1.034 (0.180)
ξ_10_	Michaelis-Menten constant	
	for HIF-1α-aOH as substrate of PHD [nM]	8.127 (1.155)
φ	FIH equilibrium level [nM]	0.829 (0.224)
α_1_, α_2_	HIF-1 mediated inhibition in mTOR regulation [nM]	1.163 (0.337),
		0.386 (0.126)

*In the right column we report the estimated parameter value, indicating mean and standard deviation (s.d.)*.

Recent results (24, 25) showed the connection between IL-15 and mTOR activity in NK cells, indicating that the AKT-mTOR pathway is indispensable for efficient cell activity and immune functions of NK cells. We therefore focused on the latter signaling pathway, neglecting other cascades [such as Ras-Raf-MEK and JAK/STAT5 (26)] which are also known to be initiated by IL-15. Further, IL-15 stimulation in neutrophils and human peripheral blood lymphocytes has been shown to activate NF-κB and STAT3 (27–29). All in all, we assumed that IL-15 (*y*_1_) activates AKT (*y*_2_), NF-κB (*y*_7_), and STAT3 (*y*_8_). For a general formulation we further assumed that IL-15 enters the system at constant rate *a*_1_ and decays at rate *d*_1_, that is, the IL-15 dynamics is given by

(2) $$\begin{array}{l} y_{1}^{\prime }\left(t\right)=a_{1}-d_{1}y_{1}. \end{array}$$

Activation of AKT is assumed to occur via IL-15 (30) (activation rate *k*_1_), other external mediators (basal activation rate *a*_2_), and also via STAT3 (maximal rate *k*_*S*_) (31). We further assumed that AKT constantly decays at rate *d*_2_, yielding

(3) $$\begin{array}{l} y_{2}^{\prime }\left(t\right)=a_{2}+k_{1}y_{1}+k_{S}\frac{y_{8}^{n_{2}}}{\xi_{28}^{n_{2}}+y_{8}^{n_{2}}}-d_{2}y_{2}. \end{array}$$

Following the literature (32) we assumed that AKT activates mTOR (*y*_3_) at rate *k*_2_. mTOR basal activation and decay rate are denoted by *a*_3_ and *d*_3_, respectively. The inhibitory effect that hypoxia has on mTOR (33) is included in the model by means of a negative feedback regulated by the HIF-1 complex (*y*_6_). Hence, for the mTOR dynamics we obtained

(4) $$\begin{array}{l} y_{3}^{\prime }\left(t\right)=\left(a_{3}+k_{2}y_{2}\right)\frac{\alpha_{1}}{\alpha_{2}+y_{6}}-d_{3}y_{3}. \end{array}$$

We denote phosphorylated STAT3 by *y*_8_. STAT3 basal activation and decay rates are *a*_8_ and *d*_8_, respectively. Both IL-15 and mTOR are known to induce phosphorylation of STAT3 (29, 34), here assumed to occur at rate *k*_6_ and *k*_8_, respectively. All in all, the differential equation for STAT3 reads

(5) $$\begin{array}{l} y_{8}^{\prime }\left(t\right)=a_{8}+k_{8}y_{3}+k_{6}y_{1}-d_{8}y_{8}. \end{array}$$

The last protein upstream of HIF-1α that we considered in our model is NF-κB, for which we assumed basal activation (at rate *a*_7_) and decay (*d*_7_). NF-κB is further activated via IL-15 (27, 28) (activation rate *k*_7_), via mTOR (35) (*k*_15_) and via the HIF-1 complex (36, 37) (*k*_14_), yielding

(6) $$\begin{array}{l} y_{7}^{\prime }\left(t\right)=a_{7}+k_{7}y_{1}+k_{14}y_{6}+k_{15}y_{3}-d_{7}y_{7}. \end{array}$$

HIF-1α mRNA basal synthesis and degradation are defined to occur at rate *a*_9_ and *d*_9_, respectively. Further, we assumed that HIF-1α mRNA is regulated by NF-κB (at rate *k*_9_) and STAT3 (at rate *k*_3_),

(7) $$\begin{array}{l} y_{9}^{\prime }\left(t\right)=a_{9}+k_{9}y_{7}+k_{3}y_{8}-d_{9}y_{9}. \end{array}$$

Following previous results (19), we assumed that asparaginyl hydroxylase FIH is at steady state (φ), whereas PHDs are upregulated by HIF-1 complex and we approximated their dynamics with quasi-steady state assumptions (see section S1 for detailed explanation). Further, we assumed that HIF-1α mRNA is translated at rate *k*_α_ and HIF-1α protein decays at rate *d*_4_. We denote by *K*_O_2__ the oxygen-dependent binding force of FIH/PHD and HIF-1α (cf. section S1). In normoxia, HIF-1α is hydroxylated via FIH (assumed at maximal rate *k*_10_) and via PHD (maximal rate *k*_13_). The dynamics of HIF-1α protein (*y*_4_) is thus given by

(8) $$\begin{array}{l} \begin{array}{ccc} y_{4}^{\prime }\left(t\right) & = & k_{\alpha }y_{9}-d_{4}y_{4}-k_{4}y_{4}y_{5}+k_{5}y_{6}-k_{13}{K_{\text{O}}}_{2}\left(\Delta y_{6}+a_{11}\right)\frac{y_{4}}{\xi_{44}+y_{4}}\\ \; & \; & \;-k_{10}{K_{\text{O}}}_{2}\varphi \frac{y_{4}}{\xi_{4}+y_{4}}+k_{11}y_{10}. \end{array} \end{array}$$

In accordance with previous studies (19, 38), we assumed that asparaginyl-hydroxylated HIF-1α (HIF-1α-aOH, here denoted by *y*_10_) can be subsequently hydroxylated via PHD and then degraded, whereas prolyl-hydroxylated HIF-1α (HIF-1α-pOH) is quickly degraded. We henceforth neglected the dynamics of HIF-1α-pOH. Further, we assumed that there is some probability for HIF-1α-aOH dehydroxylation [cf. (19)]. The resulting dynamics of HIF-1α-aOH is given by

(9) $$\begin{array}{lll} y_{10}^{\prime }\left(t\right) & = & k_{10}{K_{\text{O}}}_{2}\varphi \frac{y_{4}}{\xi_{4}+y_{4}}-k_{12}{K_{\text{O}}}_{2}\left(\Delta y_{6}+a_{11}\right)\frac{y_{10}}{\xi_{10}+y_{10}}\\ \; & \; & -k_{11}y_{10}-d_{10}y_{10}. \end{array}$$

HIF-1β (*y*_5_) is constitutively expressed by the cells (synthesis rate *a*_5_), independently of the oxygen conditions (10). In hypoxia, HIF-1α accumulates and binds to HIF-1β (at rate *k*_4_) forming the transcriptional complex HIF-1, which can dissociate (rate *k*_5_). Hence, for HIF-1β and the HIF-1 complex we obtained the equations,

(10) $$\begin{array}{l} y_{5}^{\prime }\left(t\right)=a_{5}-k_{4}y_{4}y_{5}+k_{5}y_{6}-d_{5}y_{5}, \end{array}$$

and

(11) $$\begin{array}{l} y_{6}^{\prime }\left(t\right)=k_{4}y_{4}y_{5}-k_{5}y_{6}-d_{6}y_{6}, \end{array}$$

respectively. For model calibration and comparison with collected experimental data we extended the model (2)–(11) to include DMOG or other protein inhibitors. Details are given in the Supplementary Material (section S2).

### 2.6. Numerical Simulations

For the numerical integration of the non-linear ODE system (2)–(11) and numerical simulations shown in this manuscript we used the Runge-Kutta formula (4,5) and (3,2) in MATLAB® version 9.4 [routines *ode45* and *ode23s* (39)], as well as a multistep Backward Differentiation Formula (BDF) method with variable step size and order control. The latter was implemented by mean of the solver DAESOL (40, 41) in VPLAN (42), a software for simulation, parameter estimation and optimum experimental design for non-linear processes described by differential equations.

We assumed that the initial load of IL-15 in primed cells is *y*_1_(0) = 1, whereas for unstimulated cells *y*_1_(0) = 0. Collected experimental data for HIF-1α, STAT3 and AKT were normalized with respect to measurements in untreated cells at the beginning of each experiment and used for model calibration. In setting the initial conditions, we normalized AKT, mTOR, NF-κB, STAT3, HIF-1α mRNA and HIF-1β with respect to the concentration at the beginning of each *in silico* experiment, meaning that *y*_*j*_(0) = 1, for *j* = 2, 3, 5, 7, 8, 9. The total HIF-1α level was normalized with respect to the initial measurement, corresponding to untreated cells in normoxia, hence we set *y*_4_(0) + *y*_6_(0) + *y*_10_(0) = 1. As cells were pre-cultivated in normoxia, we assumed that at the beginning of our observations (*t* = 0 h) most of HIF-1α is hydroxylated, hence, we set *y*_4_(0) = 0.05, *y*_6_(0) = 0.05 and *y*_10_(0) = 0.9.

### 2.7. Model Calibration

#### 2.7.1. Comparison With Experimental Data

For comparison with experimental data, the solution *y*(·) = *y*(·|*u, p*) of the mathematical model (1) is associated with an *m*-vector of observables,

$$\begin{array}{l} g\left(t,y\left(t\right),u,p\right). \end{array}$$

Typically it is not possible to observe all states, and in general *g*(·) is a non-linear function de-pending on the states and parameters. Given an experimental setting *u*^*ex*^(·), for each observable $g_{i}\left(t_{j}^{ex},y^{ex}\left(t_{j}^{ex}\right),u^{ex},p\right),i=1,\ldots ,m$, at measurement times $t_{j}^{ex}\in \left[t_{0},t_{f}\right],j=1,\ldots ,k^{ex}$, experimental data $\eta_{ij}^{ex}$ are collected in each experiment *ex*. Experimental measurements contain additive noise

(12) $$\begin{array}{l} \eta_{ij}^{ex}=g_{i}\left(t_{j}^{ex},y^{ex}\left(t_{j}^{ex}\right),u^{ex},p\right)+\varepsilon_{ij}^{ex}, \end{array}$$

where the errors $\varepsilon_{ij}^{ex}$ are assumed to be statistically independent and normally distributed with zero mean value and variances ${\left(\sigma_{ij}^{ex}\right)}^{2}$.

The experimental settings considered in this study present “perturbation” experiments, *ex* = 1, …, *n*_*e*_, which allow to investigate perturbations of the cellular processes from their equilibrium conditions. Each perturbation experiment corresponds to a different control *u* = *u*^*ex*^ in the mathematical model (1), and the same quantities are measured in each experiment. It is biologically reasonable to assume that an unperturbed system is at the steady state. This corresponds in our case to unstimulated NK cells (*u*^*ex*^ = *u*^0^), hence to the initial condition *y*_0_. The steady state can be mathematically determined considering the dynamics of untreated cells and setting this in equilibrium (43, 44). Hence, the initial condition of the system *y*_0_ and the model parameters must satisfy the steady state constraint,

(13) $$\begin{array}{l} 0=f\left(y_{0},u^{0},p\right). \end{array}$$

#### 2.7.2. Parameter Estimation Problem

In general, given a mathematical model (1) and experimental measurements, the goal of model calibration is to determine the model parameters in (1) from the collected data. This reduces to an optimization problem of minimizing the discrepancy between model observables and the experimental data using a particular metric (45). The weighted least squares functional is known to deliver a maximum likelihood estimate for the unknown parameters (46, 47). For the calibration of the parameters of the regulatory network (2)–(11) we used a weighted *l*_2_-norm of the measurement errors,

(14) $$\begin{array}{l} l_{2}\left(y,p\right):=\frac{1}{2}\underset{i,j,ex}{\sum }\frac{{\left(\eta_{ij}^{ex}-g_{i}\left(t_{j}^{ex},y^{ex}\left(t_{j}^{ex}\right),u^{ex},p\right)\right)}^{2}}{{\left(\sigma_{ij}^{ex}\right)}^{2}}, \end{array}$$

and further included a priori information *p*_0_ by adding a Tikhonov regularization term (48)

$$\begin{array}{l} L\left(p,p^{0},\lambda \right):=\frac{1}{2}\overset{n_{p}}{\underset{\tilde{m}=1}{\sum }}\frac{{\left(p_{\tilde{m}}-p_{\tilde{m}}^{0}\right)}^{2}}{\lambda_{\tilde{m}}^{2}}, \end{array}$$

where the vector $\lambda \in {\mathbb{R}}^{n_{p}}$ controls the amount of regularization per parameter. Moreover we incorporated additional information about parameters and states (initial conditions, steady states, etc.) in the parameter estimation problem by formulating equality constraints (49, 50). We estimated parameters by solving the following multiple experiment parameter estimation (PEP) problem

$$\begin{array}{l} \text{(PEP) }\left\{ \begin{array}{c} \;\underset{y\left(\cdot \right),p}{\text{min}}\frac{1}{2}\sum_{i,j,ex}\frac{{\left(\eta_{ij}^{ex}-g_{i}\left(t_{j}^{ex},y^{ex}\left(t_{j}^{ex}\right),u^{ex},p\right)\right)}^{2}}{{\left(\sigma_{ij}^{ex}\right)}^{2}}+\frac{1}{2}\sum_{\tilde{m}=1}^{n_{p}}\frac{{\left(p_{\tilde{m}}-p_{\tilde{m}}^{0}\right)}^{2}}{\lambda_{\tilde{m}}^{2}},\\ \;\text{s.t. }{\left(y^{ex}\right)}^{\prime }\left(t\right)=f\left(y^{ex},u^{ex},p\right),\;t\in \left[t_{0},t_{f}\right],\;y^{ex}\left(t_{0}\right)=y_{0}^{ex}\left(p\right),\\ \;r^{ex}\left(y^{ex}\left(t_{1}^{ex}\right),y^{ex}\left(t_{2}^{ex}\right),\ldots ,y^{ex}\left(t_{k}^{ex}\right),p\right)=0,ex=1,\ldots ,n_{e}. \end{array}\right. \end{array}$$

#### 2.7.3. Numerical Methods for Parameter Estimation

For least squares minimization, as those in (PEP), a frequently adopted approach is the derivative-based iterative Gauss-Newton method (45, 51). In this work we applied an “all-at-once” parameter estimation method based on a direct multiple shooting approach for dynamic systems (51) and a generalized Gauss-Newton method for optimization (50, 51). This is a boundary value problem approach, in which the system of differential Equations (1) is discretized including boundary conditions. The discretized system is treated as a non-linear constraint of the least squares objective function (52). For the multiple shooting approach, a suitable grid of multiple shooting nodes was chosen and, at each multiple shooting grid point, the values of the state variables were included as additional optimization variables. On each subinterval an additional initial value problem was solved. To maintain the continuity and feasibility of the solution, we included additional matching conditions (50, 52). The splitting of the integration interval leads to a numerically stable system (52). The resulting finite dimensional non-linear constrained least squares problem can be formally written as

(15) $$\begin{array}{l} \underset{s,p}{\text{min}}\frac{1}{2}\|F_{1}\left(s,p\right){\|}_{2}^{2},\text{ s.t. }F_{2}\left(s,p\right)=0, \end{array}$$

where the constraints *F*_2_(*s, p*) = 0 include the multiple shooting parameterization of the dynamical model and *s* denotes the vector of states in the parametrized model. The problem (15) was solved by a generalized Gauss-Newton method. At each iteration of the Gauss Newton method

$$s^{k+1}=s^{k}+t^{k}\Delta s^{k},\;p^{k+1}=p^{k}+t^{k}\Delta p^{k},\;t^{k}\in ]0,1]$$

the increments Δ*s*^*k*^, Δ*p*^*k*^ solve the linearized problem

(16) $$\begin{array}{l} \left\{ \begin{array}{c} \;\underset{\Delta s,\Delta p}{\min}\;\frac{1}{2}\|F_{1}\left(s^{k},p^{k}\right)+\frac{\partial F_{1}\left(s^{k},p^{k}\right)}{\partial s}\Delta s+\frac{\partial F_{1}\left(s^{k},p^{k}\right)}{\partial p}\Delta p{\|}_{2}^{2},\\ \text{ s.t. }F_{2}\left(s^{k},p^{k}\right)+\frac{\partial F_{2}\left(s^{k},p^{k}\right)}{\partial s}\Delta s+\frac{\partial F_{2}\left(s^{k},p^{k}\right)}{\partial p}\Delta p=0. \end{array}\right. \end{array}$$

For the case considered in this work, the linearized problem (16) shows special structures due to multiple experiments and multiple shooting approaches. These structures are efficiently exploited in a tailored linear algebra method for the solution of (16). A numerical analysis of the well-posedness of the problem and an assessment of the error of the resulting parameter estimates were performed at the solution of the problem (PEP), based on the analysis of the corresponding sensitivity (or Jacobian) matrix *J*

(17) $$\begin{array}{l} J=J\left(s,p\right)=\left(\begin{array}{c} J_{1}\left(s,p\right)\\ J_{2}\left(s,p\right) \end{array}\right)=\left(\begin{array}{cc} \frac{\partial F_{1}\left(s,p\right)}{\partial s} & \frac{\partial F_{1}\left(s,p\right)}{\partial p}\\ \frac{\partial F_{2}\left(s,p\right)}{\partial s} & \frac{\partial F_{2}\left(s,p\right)}{\partial p} \end{array}\right). \end{array}$$

Further details can be found in (49, 50). In particular, we computed the linear approximation of the variance-covariance matrix for the constrained parameter estimation problem (PEP)

(18) $$\begin{array}{l} Cov=Cov\left(s,p\right)=J^{+T}\left(\begin{array}{cc} \text{diag}{\left\{\sigma_{ij}^{ex}\right\}}_{i,j,ex} & 0\\ 0 & 0 \end{array}\right)J^{+}, \end{array}$$

and the standard deviations of states and parameters as square root of the corresponding diagonal elements of the matrix *Cov*. In (18) the matrix *J*^+^ denotes the generalized inverse of the sensitivity matrix *J*(*s, p*), that is *J*^+^*JJ*^+^ = *J*, and 0 denotes the zero matrices of the corresponding dimensions.

The above sketched methods are implemented in the software VPLAN (42), which includes the parameter estimation software PARFIT (53, 54). VPLAN was used for parameter estimation in this study.

#### 2.7.4. Initial Guesses

Before starting the actual optimization procedure (PEP), we determined initial guesses in a reasonable scale for the parameters that needed to be estimated. Good initial guesses are important for convergence of the parameter estimation methods used in this work. In certain cases prior information *p*^0^ for initial guesses is found in the literature (cf. Table 2). When results published in previous studies did not help, we applied homotopy related methods (55) and pre-estimated the parameters using Wolfram Mathematica® version 11.3 with the *Ndsolve* and *manipulate* routines, as well as DAESOL in VPLAN.

#### 2.7.5. Sensitivity Analysis

In a second step, before starting parameter estimation, we performed a local sensitivity analysis at parameter values *p*^0^ and corresponding solutions *y*^*ex*^(·|*u*^*ex*^, *p*^0^), *ex* = 1, …, *n*_*e*_ of ODE systems. The goal of the local sensitivity analysis is to find those parameters, which can be estimated reliably with the model and the given measurements. The local sensitivity analysis is performed using the sensitivity matrix *J*^0^ = *J*(*s*^0^, *p*^0^). If the sensitivity matrix *J*^0^ has almost linear dependent columns, then it is ill-conditioned and the parameter vector is badly identifiable (48, 56). We computed the singular value decomposition of the sensitivity matrix *J*^0^. The reciprocal of the minimal singular value yields the collinearity index, which, if it is large, indicates that the sensitivity matrix has almost linear dependent columns (48, 56, 57). In this work we set a minimal threshold 0.1 for rejecting small singular values and for determining the subset of parameters corresponding to almost linear independent columns of *J*^0^. This allowed to fix 13 parameters which correspond to almost linear dependent columns of *J*^0^. The remaining 25 parameters are reliably identifiable and were estimated by mean of (PEP).

### 2.8. Model Robustness

We implemented extrinsic and intrinsic stochastic perturbations using a Monte Carlo analysis. For extrinsic perturbations we varied the input stimulation IL-15 (*y*_1_(*t*)) ±25% around its original value. We measured the effect of these perturbations on total HIF-1α in the model accounting for DMOG treatment and IL-15 stimulation at the steady-state level. For intrinsic perturbations we varied specific parameters (*a*_2_, *a*_3_) and measured the effect of these perturbations on total HIF-1α in the model accounting for DMOG treatment and IL-15 stimulation at the steady state level. We implemented the intrinsic and extrinsic stochastic perturbations by varying the specific elements 25% around their original value and sampling 1000 times from a uniform distribution. The Monte Carlo analysis was implemented in Wolfram Mathematica® version 11.2.

## 3. Results

### 3.1. IL-15-induced HIF-1α Protein Accumulation in Peripheral NK Cells

**Figure 2:**
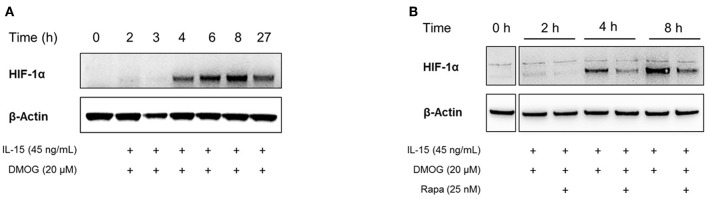
In the presence of DMOG, stimulation of NK cells with IL-15 induces the accumulation of HIF-1α protein. Whole cell extracts were used for immunoblotting of HIF-1α. Equal gel loading was confirmed by β-actin expression. **(A)** After preincubation under normoxia for 16 h, NK cells were treated with IL-15 and DMOG for indicated additional time periods. **(B)** In addition to IL-15 and DMOG, NK cells were treated with rapamycin (Rapa), which reduces HIF-1α protein accumulation.

In cells exposed to hypoxia, the stabilization and activation of HIF-1α is well characterized (58). Instead of manipulating the oxygen tension to induce HIF-1α, pharmacological inhibitors of HIF-1α hydroxylation can be used as well. We used a cell-permeable pan-hydroxylase inhibitor, DMOG, to inhibit oxygen-sensitive hydroxylases that target HIF-1α for proteasomal degradation and its transcriptional inactivation. After preincubation under normoxia for 16 h, peripheral NK cells were stimulated with the pro-inflammatory cytokine IL-15 in the presence of DMOG for different time periods. Whole cell extracts were prepared and the response of NK cells to IL-15 stimulation and DMOG treatment was assessed by evaluating the expression of HIF-1α measured by Western Blot analysis (Figure 2). The expression of β-actin was monitored to confirm equal loading.

HIF-1α expression was barely detectable in the first 3 h of IL-15 and DMOG stimulation. However, after 4 h we detected accumulation of HIF-1α protein, which further increased over 8 h and was maintained to at least 27 h, although to a lesser extent than at 8 h (Figure 2A).

IL-15 signaling in NK cells through the kinase mTOR has previously been reported to be essential for their expansion in the bone marrow and sustained activation (24). Moreover, among other signaling pathways, the PI3K/mTOR pathway has been linked to the induction of HIF-1α protein expression in immune cells, including T lymphocytes (59). To study the role of mTOR in HIF-1α protein expression in NK cells, we stimulated the cells with IL-15 and DMOG in the presence or absence of pharmacological mTOR inhibitor rapamycin. As shown in Figure 2B, mTOR inhibition reduced HIF-1α levels. Nevertheless, HIF-1α signals remained detectable, pointing to other upstream regulators of HIF-1α protein accumulation in IL-15 stimulated NK cells.

### 3.2. Model Parameters

**Figure 3:**
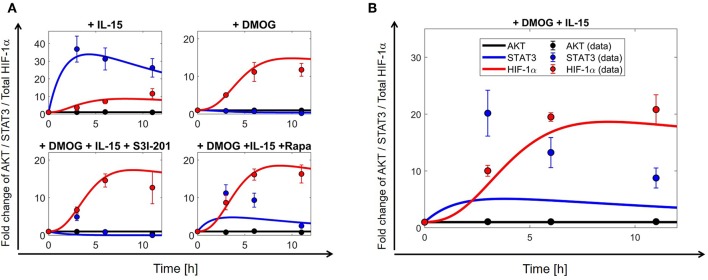
The mathematical model (2)–(11) explains collected time series for HIF-1α, STAT3, and AKT. Parameters used for numerical simulations are given in Tables 1, 2. **(A)** Model calibration results: comparison of numerical simulations (continuous curves) and collected experimental data (dots ± S.E.) of total HIF-1α (*y*_4_ + *y*_6_ + *y*_10_, red curves/dots), STAT3 (blue) and AKT (black). The model is fitted to data collected in different experimental settings: (upper left) IL-15-stimulated NK cells; (upper right) DMOG treated cells; (lower left) IL-15-stimulated NK cells treated with DMOG and STAT3 inhibitor (S3I-201); (lower right) IL-15-stimulated cells treated with DMOG and with mTOR inhibitor rapamycin (Rapa). **(B)** Model validation results: comparison of numerical simulations (continuous curves) and collected experimental data (dots ± S.E.) of total HIF-1α (*y*_4_ + *y*_6_ + *y*_10_, red curves/dots), STAT3 (blue), and AKT (black) for IL-15-stimulated cells treated with DMOG. Experimentally collected data points are reported in Table S2.

The model (2)–(11) has been calibrated on time series for AKT, STAT3 and HIF-1α collected from NK cells under different experimental conditions (Table S2), namely under stimulation/treatment with: (i) IL-15; (ii) DMOG; (iii) IL-15 + DMOG + rapamycin; (iv) DMOG + IL-15 + S3I-201. We interpret (i)–(iv) as “perturbation experiments” from the initial (equilibrium) condition. Biological sense and previous literature on mathematical modeling of cellular dynamics (43, 44) suggest that it is important to assume that untreated cells are in the steady state. Such an assumption yields 10 algebraic equations (steady state constrains) on the parameter, which together with the collected time series data of the four perturbation experiments (39 data points, cf. Table S2) were used to calibrate the model. Table 2 reports the 25 estimated parameters and Figure 3A shows the model fit. Model parameters either estimated or fixed from previous literature are reported in Tables 1, 2. With these parameter values we ran an *in-silico* experiment for NK cells stimulated with IL-15 and treated with DMOG. The obtained numerical simulations were used to validate our model, comparing the model predictions with collected experimental data for AKT, STAT3 and HIF-1α time series of NK cells stimulated with IL-15 and treated with DMOG (Figure 3B). To depict the statistical significance of the parameter estimates, Table 2 also reports the standard deviation for each estimated parameter value.

### 3.3. The Mathematical Model Explains the Dynamics of HIF-1α Accumulation

Besides the known hypoxia-induced HIF-1α stabilization and AKT-mTOR-mediated increase in protein translation, HIF-1α can also be induced through increased transcription involving activated transcription factors, among others STAT3 as shown in T lymphocytes (60) and B lymphocytes (61). To determine the role of AKT, mTOR and STAT3 as mediators of HIF-1α accumulation downstream of IL-15, we collected time series data (Figure 3, Table S2) for the phosphorylation status of AKT (Ser473) and STAT3 (Ser727), representing the activated forms of the proteins (62, 63). For optimal parameter estimation, we collected data from NK cells isolated from blood of the same donors, cultured in normoxia, chemical hypoxia (DMOG) and treated with a STAT3 or mTOR inhibitor.

The model is able to reproduce data collected for HIF-1α, STAT3 and AKT in different experimental settings (Figure 3). In particular, model predictions match time series data of HIF-1α protein expression and indicate that simultaneous exposure of NK cells to IL-15 and DMOG (Figure 3B) increases the levels of total HIF-1α, compared to HIF-1α levels in cells either stimulated with IL-15 or treated with DMOG (Figure 3A). Moreover, inhibition of mTOR or STAT3 leads to reduction of HIF-1α levels, suggesting that both proteins are involved in the regulation of IL-15 induced HIF-1α accumulation in DMOG treated cells. Model assumptions and calibration results (cf. Tables 1, 2) indicate that the external regulation of IL-15, NF-κB, STAT3, and HIF-1α-mRNA is negligible in order to explain collected time series in NK cells under the proposed experimental setting. Further, parameter estimation and model discrimination (results not shown here) suggest that DMOG reduces IL-15-mediated STAT3 activation (see section S2).

The collected data (cf. Table S2) further show that IL-15, DMOG or inhibition of either mTOR or STAT3 does not affect the levels of phosphorylated AKT (Ser473) in human NK cells. After preliminary steps in the parameter estimation procedure, we obtained $k_{1}\approx 10^{-5},k_{S}\approx 10^{-4}$ (cf. Table 1). The sensitivity analysis which followed indicated that the two values have small effects on the objective function in (PEP). This suggests that, the order of magnitude of *k*_1_ and *k*_*S*_ being much smaller than those of all other parameters, the two parameters could be set to zero without much affecting the simulation results, and the AKT dynamics in Equation (3) can be simplified and described by a linear equation,

$$\begin{array}{l} y_{2}^{\prime }\left(t\right)=a_{2}-d_{2}y_{2}. \end{array}$$

**Figure 4:**
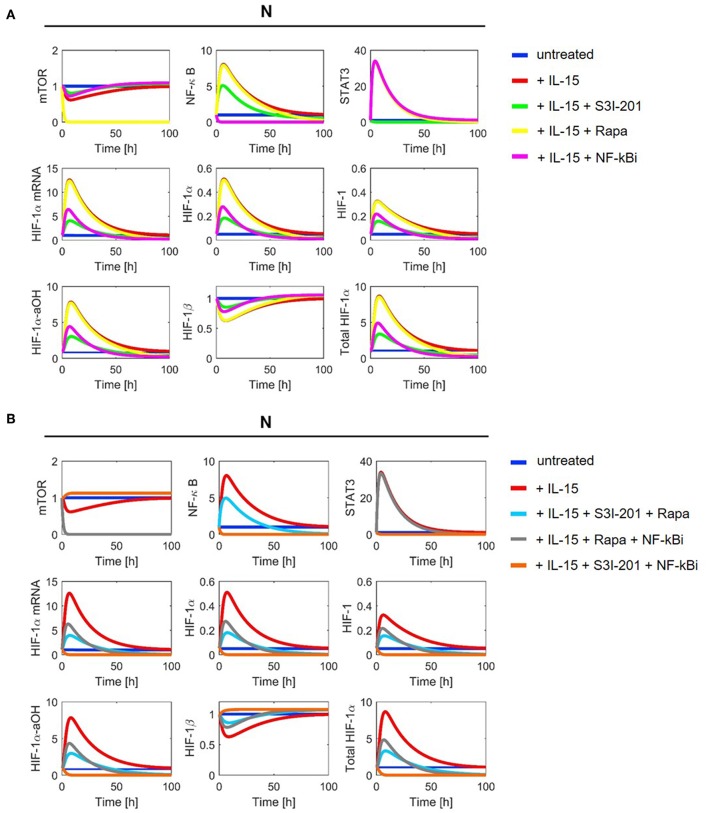
Model simulation in NK cells without DMOG (N): **(A)** with one inhibitor and **(B)** with two inhibitors. Colors indicate the following experimental settings: (blue) untreated cells; (red) cells primed with IL-15; (green) cells primed with IL-15 and treated with STAT3 inhibitor S3I-201; (yellow) cells primed with IL-15 and treated with mTOR inhibitor rapamycin (Rapa); (magenta) cells primed with IL-15 and treated with NF-κB inhibitor (NF-κBi); (cyan) cells primed with IL-15 and treated with STAT3 inhibitor and rapamycin; (gray) cells primed with IL-15 and treated with NF-κB inhibitor and rapamycin; (orange) cells primed with IL-15 and treated with NF-κB and STAT3 inhibitors.

**Figure 5:**
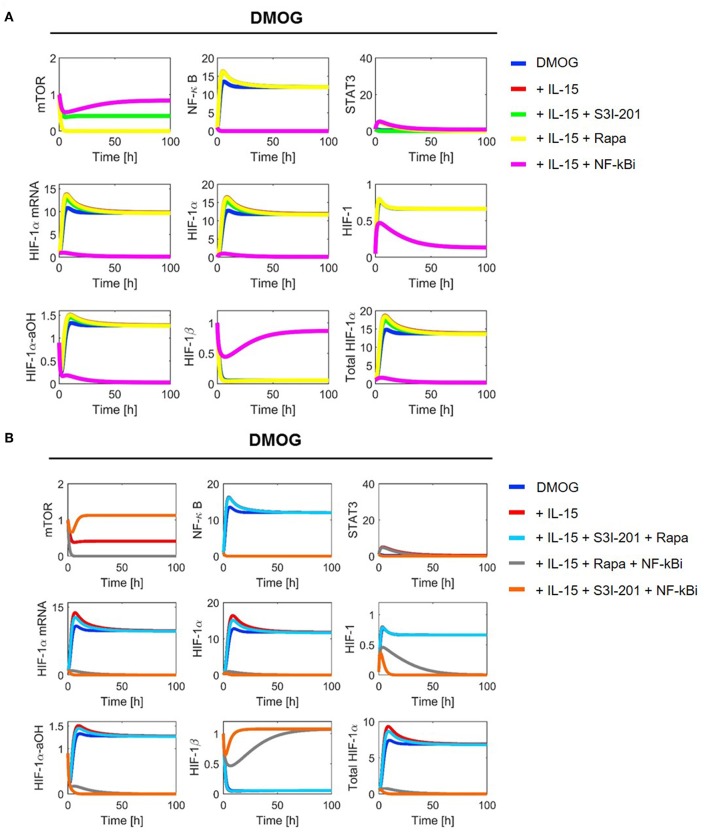
Model simulation in chemical hypoxia (20 μM DMOG): **(A)** with one inhibitor and **(B)** with two inhibitors. Colors indicate the following experimental settings: (blue) untreated cells; (red) cells primed with IL-15; (green) cells primed with IL-15 and treated with STAT3 inhibitor S3I-201; (yellow) cells primed with IL-15 and treated with mTOR inhibitor rapamycin (Rapa); (magenta) cells primed with IL-15 and treated with NF-κB inhibitor (NF-κBi); (cyan) cells primed with IL-15 and treated with STAT3 inhibitor and rapamycin; (gray) cells primed with IL-15 and treated with NF-κB inhibitor and rapamycin; (orange) cells primed with IL-15 and treated with NF-κB and STAT3 inhibitors.

With parameter values as indicated in Tables 1, 2 we ran numerical simulations of model (2)–(11) for NK cells under different experimental conditions. Figures 4, 5 show the simulation results for all^1^ model components in normoxia without (N, O_2_ = 21%) and with DMOG (20 μM, chemical hypoxia), treated with one (A panels) or two inhibitors at the same time (B panels). The level of total HIF-1α, which we define as the sum of HIF-1α protein, HIF-1 complex and HIF-1α-aOH, is significantly higher in DMOG treated cells than in untreated cells in normoxia. The major component of the sum in DMOG treated cells is HIF-1α, whereas its hydroxylated form predominates in normoxic cells. As expected, we observe that HIF-1β is stable in normoxia. However, in the presence of available HIF-1α, HIF-1β is consumed for HIF-1 complex formation.

Consistent with the model assumptions, we observe mTOR inhibition by rapamycin. Moreover, the stabilization of HIF-1α in DMOG treated cells and the subsequent formation of HIF-1 results in a negative feedback on mTOR (Figure 5). NF-κB shows higher activity in DMOG treated cells compared to untreated cells and our simulations predict an essential role for NF-κB as a regulator of HIF-1α-mRNA and protein in DMOG treated cells. In contrast, the IL-15-induced STAT3 activity is higher in cells without DMOG and inhibition of STAT3 results in an important reduction of HIF-1α-mRNA and protein levels. Combined inhibition of both transcription factors abolishes HIF-1α enrichment in both, DMOG treated and untreated cells.

### 3.4. Regulators of HIF-1α Enrichment

Parameter values used for data fitting (Table 1) in Figure 3A indicate that the external regulation rates of IL-15, mTOR, and STAT3 are small or negligible in the considered cell cultures. Nevertheless, such parameters could change from donor to donor, in particular if affected by inflammatory conditions or cancer (5, 64).

**Figure 6:**
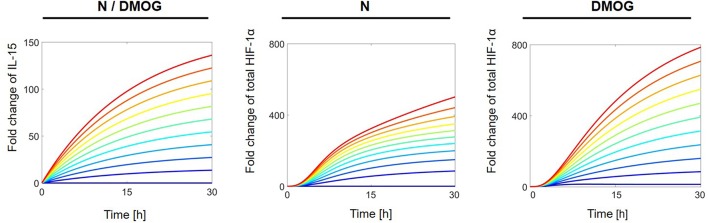
Effects of external IL-15 regulation on total HIF-1α in NK cells cultivated in the absence (N) or presence of DMOG. External IL-15 regulation rate (*a*_1_) is varied in [0, 10] nM h^−1^ with regular steps. Other parameters and initial values are fixed as in Tables 1, 2. Curves with same color correspond to the same parameter value and follow the jet color map in MATLAB®, with dark blue corresponding to the lowest value (*a*_1_ = 0 nM h^−1^) and red to the highest value (*a*_1_ = 10 nM h^−1^). Left: IL-15 dynamics is equal in both, NK cells cultivated without and with DMOG as there is no feedback on IL-15 in the model (cf. Equation 2); middle: Total HIF-1α in untreated NK cells; right: Total HIF-1α in NK cells cultivated with DMOG.

To investigate the influence of external regulators on the behavior of the total HIF-1α stabilization, we systematically perturbed the constant activation rate of different proteins in the network. First we studied the effect of external regulation of IL-15 on the stabilization of total HIF-1α in normoxia in the presence or absence of DMOG. For this we simulated ideal experiments in which the cells are exposed to continuous stimulation. We ran computer simulations varying the IL-15 external regulation parameter *a*_1_ in the interval [0, 10] nM h^−1^ and plotted the solution of total HIF-1α over time. All other parameter values as well as initial conditions were fixed as indicated in Materials and Methods. Figure 6 shows the results for IL-15 (left), and for total HIF-1α in the absence (middle) or presence of DMOG (right), with dark blue curves corresponding to the lowest value (*a*_1_ = 0 nM h^−1^) and red curves to the highest value (*a*_1_ = 10 nM h^−1^). Simulations confirm the synergistic effect of IL-15 and DMOG treatment on HIF-1α. The stronger the continuous IL-15 stimulus, the higher are the total HIF-1α levels. Compared with untreated cells, HIF-1α levels reach two to three times higher steady state^2^ values in the presence of DMOG.

**Figure 7:**
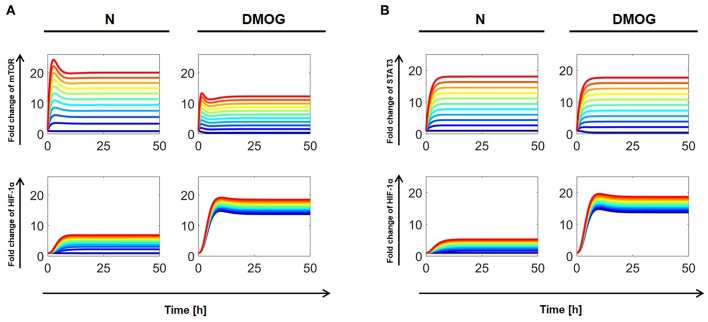
**(A)** Effects of external mTOR regulation on total HIF-1α in NK cells without (N) or with DMOG. External mTOR activation rate (*a*_3_) is varied in [0, 10] nM h^−1^ with regular steps. **(B)** Effects of external STAT3 regulation on total HIF-1α. External STAT3 activation rate (*a*_8_) is varied in [0, 10] nM h^−1^ with regular steps. All other parameters and initial values are fixed as in Tables 1, 2. Curves with same color correspond to the same parameter value and follow the jet color map in MATLAB®, with dark blue corresponding to the lowest value (*a*_*j*_ = 0 nM h^−1^, *j* = 3, 8) and red to the highest value (*a*_*j*_ = 10 nM h^−1^, *j* = 3, 8).

Similarly, we investigated the dependence of total HIF-1α accumulation on the external regulation of mTOR and STAT3. We considered cells with or without DMOG and proceeded as above by varying the parameters *a*_3_ (for mTOR) and *a*_8_ (for STAT3) in the interval [0, 10] nM h^−1^. For investigations on the steady state of the systems (*t* = 100 h), it makes no difference if cells are initially stimulated with IL-15 or not, as the effect of initial stimulation has vanished at the steady state (cf. Figures 4, 5). The results are shown in Figure 7, where dark blue curves correspond to the lowest value (*a*_*j*_ = 0 nM h^−1^, *j* = 3, 8) and red curves to the highest value (*a*_*j*_ = 10 nM h^−1^, *j* = 3, 8). Our computer simulations confirm that higher HIF-1α concentration in DMOG treated cells induces a negative feedback and downregulates mTOR (Figure 7A). As Figure 7B suggests, increasing STAT3 external regulation leads to higher HIF-1α levels, which are amplified by DMOG treatment, although STAT3 levels in DMOG treated cells are slightly lower than in cells without DMOG.

**Figure 8:**
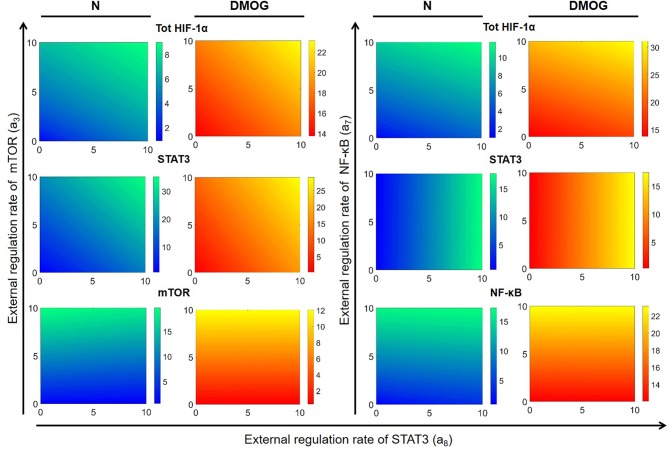
Effects of external regulation of mTOR, NF-κB, and STAT3 in NK cells without (N) or with DMOG. External activation rates for mTOR (*a*_3_) and STAT3 (*a*_8_) are varied in the interval [0, 10] nM h^−1^ and for NF-κB (*a*_7_) in the interval [10, 20] nM h^−1^. Other parameters and initial values are fixed as in Tables 1, 2. Steady states (100 h) of the model solutions are computed for total HIF-1α (first row), STAT3 (second row), and mTOR or NF-κB (third row).

**Figure 9:**
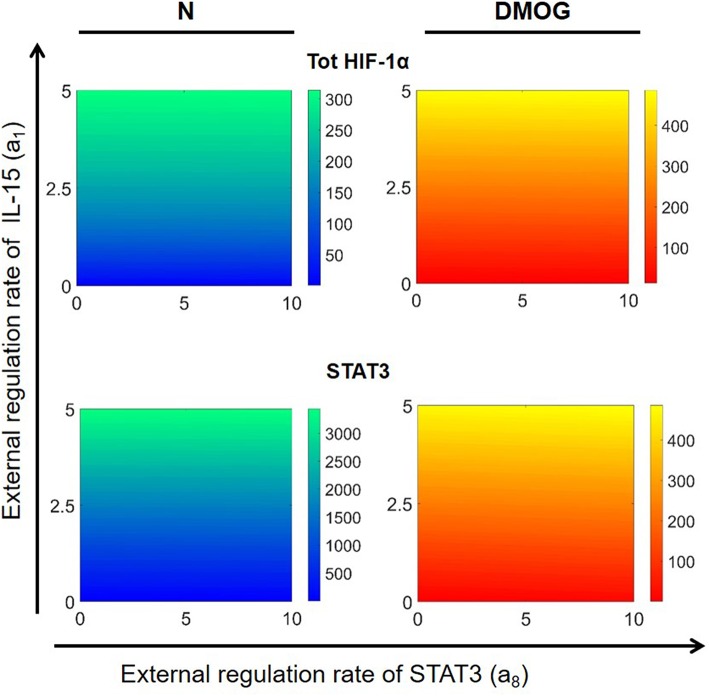
Effects of external regulation of IL-15 (*a*_1_) and STAT3 (*a*_8_) in NK cells without DMOG (N, left) and DMOG treated cells (right). External regulation rates are varied in the intervals [0, 5] nM h^−1^ for IL-15 and [0, 10] nM h^−1^ for STAT3. Other parameters and initial values are fixed as in Tables 1, 2. Steady states (100 h) of the model solutions are computed for total HIF-1α (first row) and STAT3 (second row).

We further investigated the dependence of total HIF-1α enrichment on two signals at the same time. We started by varying the external activation rate of mTOR (*a*_3_) and STAT3 (*a*_8_) in the interval [0, 10] nM h^−1^, ran simulations up to *t* = 100 h and obtained numerical solutions of the mathematical model at the steady state. Figure 8 shows the results for total HIF-1α, STAT3, and mTOR in NK cells cultivated with or without DMOG. The same figure shows also the effect of simultaneous changes in the external activation rate of NF-κB (*a*_7_ in the interval [0, 10] nM h^−1^) and STAT3 (*a*_8_ in the interval [0, 10] nM h^−1^). Again, we observe a central role of STAT3 as regulator of HIF-1α enrichment, especially in synergy with NF-κB. An increase in STAT3 external regulation rate combined with an increase in the external regulation rate of NF-κB leads to a higher amplification of total HIF-1α compared to STAT3 combined with mTOR. Plots in Figure 8 also reflect the negative feedback of induced HIF-1 on mTOR: (i) in general, NK cells treated with DMOG have lower levels of activated mTOR than untreated cells and (ii) higher concentration of activated STAT3 (due to increasing *a*_8_ rate) induces HIF-1α, resulting in higher levels of HIF-1 complex, which in turn is known to inhibit mTOR. Finally, Figure 8 stresses the role of HIF-1 as activator of NF-κB. In normoxic cells, where HIF-1 levels are low, NF-κB activity is low, despite increasing of external regulation. In contrast, in DMOG treated cells, HIF-1 accumulation leads to upregulation of NF-κB. This is in accordance with data obtained in neutrophils demonstrating that NF-κB is an important downstream effector of the HIF-1α-dependent response (37). Figure 9 shows HIF-1α steady states in dependence on the external regulation rate of IL-15 (*a*_1_ varying in [0, 10] nM h^−1^) and activation of STAT3 (*a*_8_ varying in the interval [0, 10] nM h^−1^). The results confirm the synergistic effect of IL-15, STAT3 and DMOG in increasing HIF-1α levels.

**Figure 10:**
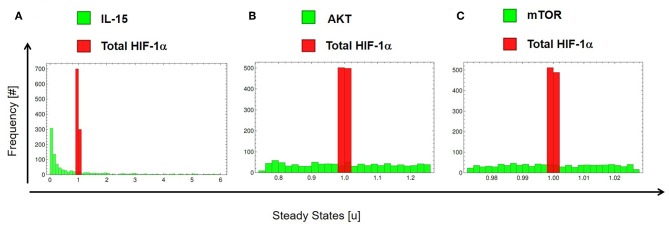
Stochastic extrinsic and intrinsic perturbations of the HIF-1α regulatory network in IL-15 stimulated cells treated with DMOG. Histograms accounting for total HIF-1α relative steady state variations after stochastically varying **(A)** IL-15, **(B)** the AKT basal activation rate (*a*_2_), and **(C)** the mTOR basal activation rate (*a*_3_). The model is robust to internal and external stochastic perturbations, indicating that variation of ± 25% in the above elements result in minimal variations (<10%) in the steady state of total HIF-1α.

Besides the above deterministic perturbations, we tested the network robustness with a stochastic approach. Robustness allows a system to maintain its function, regardless of external and internal perturbations (65). We perturbed specific elements of the system 25% around their originally estimated value (parameter values are otherwise fixed as in Tables 1, 2). We applied stochastic perturbations using the Monte Carlo method (see section 2.8) and computed how external (in IL-15) and internal (in mTOR and AKT) changes affect the steady state of total HIF-1α in IL-15 stimulated cells treated with DMOG. Figure 10 shows the histograms for IL-15 (A), AKT (B), and mTOR (C) (green) and total HIF-1α (red). Our results show that the model is robust to internal and external stochastic perturbations, indicating that variations of ± 25% in IL-15, in the AKT activation rate *a*_2_ ± 25% or in the mTOR activation rate *a*_3_ ± 25% result in minimal variations (<10%) in the steady state of total HIF-1α.

**Figure 11:**
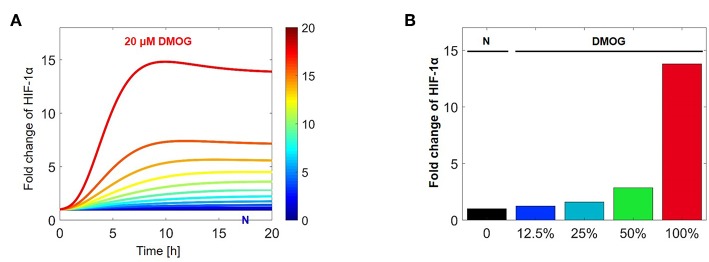
Stabilization of HIF-1α in dependence on DMOG concentration after IL-15 stimulation. **(A)** Evolution of HIF-1α in time, depending on the DMOG dosage with dark blue corresponding to no DMOG (N) and red corresponding to 20 μM DMOG. In these simulations cells are initially stimulated with IL-15. **(B)** Fold change of HIF-1α stabilization at the equilibrium (*t* = 100 h). Simulation of NK cell treatment with increasing concentrations of DMOG. Parameter values for these simulations are chosen as in Tables 1, 2, with 100% corresponding to 20 μM DMOG.

With the help of numerical simulations we tested how HIF-1α stabilization is affected by increasing concentration of DMOG. Assuming that NK cells are treated with DMOG and stimulated with IL-15 at time *t* = 0 h, we changed the DMOG concentration from 0 to 100%, with 100% corresponding to 20 μM. Figure 11A shows the evolution of HIF-1α in time, with HIF-1α stabilization depending on the DMOG dosage. We computed the fold change of HIF-1α stabilization at the equilibrium (*t* = 100 h) and compared control cells (untreated) with cells treated with different DMOG concentrations (Figure 11B). The relation between HIF-1α stabilization and DMOG dosage is non-linear and doubling the DMOG dose does not lead to twice as high HIF-1α levels. Our results suggest an exponential trend in the relation between PHD/FIH inhibitor DMOG and HIF-1α stabilization, which is in accordance with what was previously observed for HEK cells (19).

### 3.5. Which Timing for Cell Treatment?

**Figure 12:**
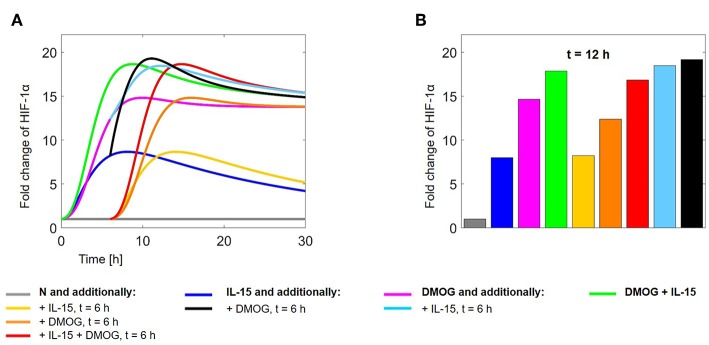
**(A)** HIF-1α evolution in time and **(B)** fold change at *t* = 12 h, normalized with respect to HIF-1α levels in untreated cells. Colors correspond to the following experimental settings: (gray) untreated cells (N) for 30 h; (dark blue) IL-15 stimulation at time *t* = 0 h; (magenta) cells in DMOG for 30 h; (green) cells in DMOG for 30 h with IL-15 stimulation at *t* = 0 h; (yellow) IL-15 stimulation at *t* = 6 h; (orange) DMOG treatment at *t* = 6 h; (red) IL-15 stimulation and DMOG treatment at *t* = 6 h; (light blue) DMOG treatment at *t* = 0 h and IL-15 stimulation at *t* = 6 h; (black) IL-15 stimulation at *t* = 0 h and DMOG treatment at *t* = 6 h.

Model (2)–(11) can be used for a number of *in silico* experiments to test the validity of biological hypotheses or predict the outcome of laboratory tests. In this study we were particularly interested in the synergy of IL-15-stimulation and DMOG treatment in the stabilization of HIF-1α, already observed in the results described above. In all previous simulations, normoxic NK cells were stimulated at the beginning of the observation with IL-15 in the presence or absence of DMOG. To understand how the timing of the treatments affects HIF-1α stabilization in NK cells, we also simulated different possibilities for the timing of cell treatment combining chemical hypoxia and stimulation with IL-15 (Figure 12).

We compared the HIF-1α dynamics for the following *in silico* experiments: (gray) untreated NK cells (N) cultivated for 30 h; (dark blue) IL-15 stimulation at *t* = 0 h; (magenta) DMOG treatment for 30 h; (green) DMOG treatment for 30 h, with IL-15 stimulation at *t* = 0 h; (yellow) IL-15 stimulation at *t* = 6 h; (orange) DMOG treatment starting at *t* = 6 h; (red) IL-15 stimulation at *t* = 6 h and DMOG treatment starting at *t* = 6 h; (light blue) DMOG treatment for 30 h with IL-15 stimulation at *t* = 6 h; (black) IL-15 stimulation at *t* = 0 h and DMOG treatment starting at *t* = 6 h.

Figure 12A shows the time evolution of HIF-1α stabilization over 30 h. We observe the impulses at *t* = 6 h due to changes in the experimental conditions. On the long term, the effect of IL-15 stimulation vanishes and HIF-1α levels converge to those reached in unstimulated cells. Figure 12B shows the fold change of HIF-1α at *t* = 12 h. Values are normalized with respect to HIF-1α in untreated cells (N, gray bar). We observe that on the short time scale the timing of treatments importantly affects HIF-1α stabilization. In particular, treating the cells first with DMOG or first stimulating them with IL-15 is not equivalent (compare the black bar and the light blue bar). The highest HIF-1α levels after 12 h are reached when cells are first stimulated with IL-15 at *t* = 0 and treated with DMOG at *t* = 6 h (black bar). Cultivating cells in normoxia and treating them with IL-15 and DMOG at time *t* = 6 h (red) yields lower HIF-1α values than 12 h cultivation in the presence of DMOG after initial (*t* = 0 h) IL-15 stimulation (green).

## 4. Discussion

Being an essential mediator of cellular adaptation to hypoxia (66, 67), HIF-1α plays a critical role as regulator of inflammation and immune system response (36, 68). The understanding of its regulation is crucial in immunology.

While HIF-1α hydroxylation and degradation pathways have been recently described using mathematical methods (19, 20), less is known concerning the mechanistic description of processes regulating the levels of HIF-1α mRNA and protein (10). In this work we have presented a combined approach of experimental and mathematical analysis to understand HIF-1α regulation in human NK cells, in particular simulating hypoxic (DMOG) and inflammatory (IL-15) conditions. To the best of our knowledge, there is no previous interdisciplinary approach describing the interplay of hypoxia and IL-15 stimulation, and their effects on HIF-1α dynamics in immune cells. The proposed mathematical model (2)–(11) and the estimated parameter values (Tables 1, 2) explain collected time series for HIF-1α, also catching the dynamics of other regulatory proteins (Figure 3). Our simulation results and *in silico* experiments highlight the synergy of IL-15 and hypoxia in HIF-1α stabilization, suggesting an important role for STAT3 and NF-κB as regulators of IL-15 induced HIF-1α enrichment in peripheral NK cells.

The mathematical model proposed in this work aimed at the qualitative mechanistic description of IL-15 induced biochemical processes regulating HIF-1α stabilization in NK cells. We made use of collected time series (HIF-1α, AKT, and STAT3) for quantitative investigation, data fitting and model predictions. A limitation of our results is that model predictions for quantities lacking experimental information (e.g., NF-κB, mTOR, and HIF-1α-mRNA) can be made only on a relative scale (43). While the calibration (Figure 3A) and validation results (Figure 3B) for HIF-1α are overall very satisfactory, the quantitative match for STAT3 in cells treated with DMOG and IL-15 (Figure 3B) could be improved. This might be achieved by refining the fit for STAT3 time series in IL-15-stimulated NK cells treated with DMOG and rapamycin (Figure 3A). In this study, we performed an all-at-once parameter estimation, applying a direct multiple shooting approach and a gradient-based (generalized Gauss-Newton) method (cf. section 2.7). The method is known to perform well and converge fast [cf. (45)] but, being a local optimization method, it might get stuck in a local optimal minimum. A possible method to overcome local minima is to perform many independent optimization runs starting from randomly selected starting points (69). Alternatively, one could adopt global optimization methods, which however can be computationally very costly (45, 69).

Concerning the statistical significance of the parameter estimates (Table 2) we have adopted here a first order approximation of non-linear confidence regions. Parameter estimation and identifiability could be further refined and investigated, e.g., performing a second-order analysis of the non-linear confidence regions [cf. (70)] or exploiting the profile likelihood, as suggested by Raue et al. (69).

Our model captures essential features of HIF-1α regulation, making a number of simplifying assumptions. Several model extensions and refinements could be proposed. For example, we could include further steps in the degradation pathway of HIF-1α, as proposed by others (10, 19). Moreover, the dynamics of IL-15 is simply given by constant production and degradation rates [as it has previously been assumed by other authors, e.g., for IL-21 dynamics (71)], and sensitivity analysis indicates that the AKT dynamics is approximatively linear in the considered experimental setting (section 3.3). The reaction cascade downstream of IL-15 involves several components, including the IL-15Rβγ-subunits (4), which are known to be constitutively expressed on NK cells (72) but were neglected in the proposed mathematical model. Further, the IL-15-induced activation of AKT, NF-κB and STAT3 is modeled by means of linear terms. One possible model extension would include non-linear terms (Michaelis-Menten or higher order Hill functions) for the activation of IL-15 regulated proteins. Factors connected to IL-15 stimulations, such as IL-15 receptor binding and trafficking or other IL-15 induced signaling cascades (JAK/STAT5, Ras-Raf-MEK), might affect NK cell response to this cytokine and could also be taken into consideration. Further, the relation between mTOR and the HIF-1 complex could be investigated in detail. We have assumed that hypoxia downregulates mTOR (33) by means of a negative feedback of HIF-1 on mTOR. Nonetheless, the regulatory mechanism of mTOR is far more complicated, involving REDD1 and the Tsc1/Tsc2 complex (73). Our experimental data and modeling results show that HIF-1α accumulation in cells stimulated with IL-15 and treated with DMOG correlates with reduction of STAT3 activity. Our modeling approach suggests (cf. section S2) that the known negative feedback of HIF-1 on mTOR (33) is amplified by a direct inhibitory effect of DMOG on IL-15-induced STAT3 activation. This means that the observed STAT3 inhibition could not only be due to chemical hypoxia, stabilizing HIF-1α, but also due to additional effects of DMOG on NK cells. To further explore the role of DMOG on IL-15-induced STAT3 activation in NK cells, the experiments proposed in this study could be performed in cells cultivated in hypoxia (1% O_2_) instead of chemical hypoxia.

Spatial effects could also be taken into account for model refinement. In contrast to previous studies (19, 74), in our modeling approach we did not make any distinction between proteins in the cell cytoplasm and the nucleus, but simply consider total cellular concentrations. In general, increased model complexity necessarily calls for more detailed experimental data in order to achieve adequate model calibration and trustworthy predictions.

We hypothesize that the proposed regulatory network is appropriate for describing HIF-1α regulation not only in NK cells but also in other types of immune cells. Moreover, the model (2)–(11) can be applied to refine and extend mathematical models in which HIF-1α dynamics is involved, e.g., models of cell cycle regulation (75) or cell proliferation (76). When studying the effects of biochemical signaling at the cellular level, it might be convenient to adopt simpler regulatory networks than those proposed here [see for example (77) for a model for proliferation of IL-15 stimulated NK cells]. To this extent model reduction could be performed by mean of biological assumptions, e.g., via quasi-steady state approximations. Further, (global) sensitivity analysis results could be used to rank the relative influence of the model parameters on the model output, and could suggest how to simplify the regulatory network, identifying parameters that minimally impact model outputs [cf. (78, 79)].

Being involved in cytokine expression, myeloid cell migration and effector functions, HIF-1α regulates both innate and adaptive immunity (80). Understanding the molecular mechanisms involved in the regulation of the HIF-1α pathway, in particular in immune cells, is of central importance to the immune cell function and could be a promising strategy in the design of treatments for human inflammatory diseases and cancer. Our results indicate that NF-κB and STAT3 are important regulators of HIF-1α enrichment in IL-15 stimulated NK cells. It is tempting to speculate that a secondary effect of pharmacological STAT3 inhibition in cancer therapy may consist in a reduction of IL-15 dependent HIF-1α enrichment in NK cells, which may be expected to improve NK cell anti-tumor activity (12, 13).

## Data Availability Statement

All datasets generated for this study are included in the manuscript/Supplementary Files.

## Ethics Statement

The study was reviewed and approved by the Medical Ethics Commission II of the Medical Faculty Mannheim, Heidelberg University (2014-500N-MA).

## Author Contributions

MB and HL: conceptualization. AC, MB, EK, and HL: methodology. SV: NK cell characterization. AB, MB, and AF: software. AF: robustness analysis. AC and MB: investigation and data curation. MB and AC: writing. AC, MB, SV, MT, and HL: review and editing (original manuscript). MB, AC, AB, AF, EK, and H-GB: review and editing (revision). All authors approved the final version of the manuscript.

### Conflict of Interest

The authors declare that the research was conducted in the absence of any commercial or financial relationships that could be construed as a potential conflict of interest.

## Footnotes

^1^The dynamics of IL-15 is trivial and neither affected by oxygen saturation nor other inhibitors (in the model assumptions there is no feedback on IL-15, cf. Equation 2), hence it is equivalent in all experimental conditions and omitted in Figures 4, 5. The same holds for the AKT dynamics in view of the results of the sensitivity analysis.

^2^A complete analytical study of the steady states of system (2)–(11) and their stability was beyond the scope of this manuscript. In general, the non-linear system (2)–(11) might have, in certain parameter ranges, multiple biologically relevant steady states. However, the parameter values used for the numerical simulations in this work guarantee convergence to a unique non-negative steady state which is shifted to higher/lower values when different stimuli are considered (cf. simulations shown in Figures 4–10).
