# Supplementary material for: Interleukin-15 Signaling in HIF-1α Regulation in Natural Killer Cells, Insights Through Mathematical Models
Source: Front Immunol. 2019 Oct 16;10:2401. doi: 10.3389/fimmu.2019.02401 (PMC6805776; doi:10.3389/fimmu.2019.02401)
Supplement: Supplementary file 2 [file Data_Sheet_2.pdf]

Supplementary Material: Interleukin-15 signaling in HIF-1α regulation in natural killer cells, insights through mathematical models*

SUPPLEMENTAL DATA

The Supplementary Material contains:

Section S1: Modeling HIF-1α hydroxylation.

Section S2: Modeling inhibitors.

Table S1: Description of model variables.

Table S2: Table with collected experimental data used in this study.

S1 Modeling HIF-1α Hydroxylation

In normoxia, HIF-1α protein is hydroxylated through the hydroxylases FIH and PHD, the latter leading to degradation of HIF-1α via the Von Hippel-Landau protein (not considered in our model). Following previous work [4, 1], in the mathematical model (2)–(11) we assumed that FIH is at steady state, $\text{FIH}(t) = \varphi$ for all $t \geq 0$, whereas PHD is upregulated by the HIF-1 complex (y_6),

$$\text{PHD}'(t) = a_P + k_P y_6(t) - d_P \text{PHD}(t).$$

With quasi-steady state approximation, the dynamics of PHD in dependence of $y_6(t)$ reads

$$\overline{\text{PHD}} = a_{11} + \Delta y_6(t),$$

where the parameters are $a_{11} = a_P/d_P$ and $\Delta = k_P/d_P$. With the last relation we obtained from the literature [4, 1] the values of a_{11} and Δ as in Table 1 and Table 2.

The activity of hydroxylases is reduced in hypoxia or in hypoxia mimicking conditions (DMOG), inducing HIF-1α accumulation. In the model, we included the oxygen-dependent activity of hydroxylases by means of the oxygen binding force, K_{O_2}. This is defined as a function of the O_2 tension in the medium (x),

$$K_{\text{O}_2}(x) := \frac{x^n}{x^n + \text{O}_{2\,crit}^{\,n}}. \tag{S1}$$

Comparison with previous literature [6, 2, 3, 5] suggests that $\text{O}_{2\,crit} \approx 4\%$. With $n = 2$ we have $K_{\text{O}_2}(21\%) \approx 0.96$ and $K_{\text{O}_2}(1\%) \approx 0.06$, which is comparable with parameters used previously [4].

*Authors: Anna Coulibaly, Anja Bettendorf, Ekaterina Kostina, Ana Sofia Figueiredo, Sonia Y. Velásquez, Hans-Georg Bock, Manfred Thiel, Holger A. Lindner, Maria Vittoria Barbarossa (barbarossa@uni-heidelberg.de)

S2 Modeling Inhibitors

DMOG mimics natural hypoxia by inhibiting FIH and PHD activity thereby preventing HIF-1α hydroxylation. In the model we included the term $(1 - \rho_6 D)$ to reduce the oxygen dependent FIH/PHD binding force K_{O_2} in the presence of DMOG (D). Accordingly, the HIF-1α equation (8) is modified as follows:

$$y_4'(t) = k_\alpha y_9 - d_4 y_4 - k_{13} K_{O_2} (\Delta y_6 + a_{11})(1 - \rho_6 D) \frac{y_4}{\xi_{44} + y_4}$$
$$- k_4 y_4 y_5 + k_5 y_6 - k_{10} K_{O_2} \varphi (1 - \rho_6 D) \frac{y_4}{\xi_4 + y_4} + k_{11} y_{10}.$$

We set the parameter $D = 1$ when NK cells are treated with 20 µM DMOG. The constant ρ_6 is included to model the efficacy of DMOG as PHD/FIH inhibitor and is taken from previous literature ([4], see Table 1). Similarly, we modified equation (9).

Rapamycin has been shown to inhibit mTOR activity and its signaling pathway. In modeling the mTOR activity we assumed that this is inhibited as soon as cells are treated with rapamycin and modified equation (4),

$$y_3'(t) = (a_3 + k_2 y_2) \frac{\alpha_1}{\alpha_2 + y_6} (1 - R) - d_3 y_3, \tag{S2}$$

setting the parameter $R = 1$ when cells were treated with 25 nM rapamycin. Analogously, we modified equation (6) when we simulate cell treatment with an inhibitor of NF-κB.

S3I-201 inhibits STAT3 by blocking its phosphorylation and dimerization events necessary for activation of the protein. We described the effect of S3I-201 on STAT3 modifying the STAT3 activation rate in equation (5) by means of the factor $(1 - \rho_3 S_3)$, where S_3 represents the STAT3 inhibitor. Further, parameter estimation and preliminary model discrimination (results not shown here) suggested that treatment with DMOG reduces IL-15-mediated STAT3 activation. In order to reproduce the combined usage of S3I-201 and DMOG we have modified equation (5) as follows:

$$y_8'(t) = (a_8 + k_8 y_3 + k_6 (1 - \rho_4 D) y_1)(1 - \rho_3 S_3) - d_8 y_8. \tag{S3}$$

Hereby we fixed $D = 1$ when cells were treated with 20 µM DMOG, and $S_3 = 1$ when cells were treated with 200 µM S3I-201. The parameter ρ_3 indicates the efficacy of S3I-201 as STAT3 inhibitor in NK cells and the parameter ρ_4 the inhibitory effect of DMOG on IL-15-mediated STAT3 activation. Both parameters were estimated from experimental data (see Table 1).

REFERENCES

[1]Z. Fábián, C. T. Taylor, and L. K. Nguyen. Understanding complexity in the HIF signaling pathway using systems biology and mathematical modeling. *J. Mol. Med.*, 94(4):377–390, 2016.

[2]S. Galbán and M. Gorospe. Factors interacting with HIF-1α mRNA: novel therapeutic targets. *Curr. Pharm. Des.*, 15(33):3853–3860, 2009.

Symbol	Description [Unit]	Initial Value ($y_j(0)$)
t	Time [h]	0
$y_1(t)$	IL-15 concentration at time t [nM]	0 or 1
$y_2(t)$	AKT concentration at time t [nM]	1
$y_3(t)$	mTOR concentration at time t [nM]	1
$y_4(t)$	HIF-1α concentration at time t [nM]	0.05
$y_5(t)$	HIF-1β concentration at time t [nM]	1
$y_6(t)$	HIF-1 complex concentration at time t [nM]	0.05
$y_7(t)$	NF-κB concentration at time t [nM]	1
$y_8(t)$	STAT3 concentration at time t [nM]	1
$y_9(t)$	HIF-1α mRNA concentration at time t [nM]	1
$y_{10}(t)$	HIF-1α-aOH concentration at time t [nM]	0.9

Table S1. Variables used in the mathematical model (2)–(11), with description and initial values used in the simulations. The initial IL-15 level $y_1(0)$ is assumed to be 0 in unstimulated cells, or 1 in cells stimulated with IL-15. Variables y_2, y_3, y_7, y_8, indicate the phosphorylated form of the respective proteins.

Experiment	Time [h]	HIF-1α	STAT3	AKT
Untreated cells	0	1± 0.01	1± 0.01	1± 0.01
(i) + IL-15	3	3.63 ± 1.52	36.90 ± 7.38	1.03 ± 0.21
	6	7.17 ± 1.27	31.29 ± 6.26	1.17 ± 0.23
	11	11.58 ± 2.85	26.24 ± 5.25	0.96 ± 0.19
(ii) + DMOG	3	5.05 ± 0.06	0.83 ± 0.17	0.85± 0.17
	6	11.19 ± 2.53	0.70± 0.14	0.98 ± 0.20
	11	11.76 ± 1.7	0.21± 0.04	0.96± 0.19
(iii) + DMOG + IL-15 + rapamycin	3	8.67 ± 1.9	11.19 ± 2.24	0.80 ± 0.16
	6	16.13 ± 1.50	9.33 ± 1.866	1.09± 0.22
	11	16.30 ± 2.40	2.49 ± 0.50	0.79 ± 0.16
(iv) + DMOG + IL-15 + S3I-201	3	6.72 ± 0.81	4.88 ± 0.98	0.96 ± 0.19
	6	14.57 ± 1.75	0.90 ± 0.18	0.87 ± 0.17
	11	12.67 ± 4.29	n.d.	0.38 ± 0.08
(v) + DMOG + IL-15	3	10.03 ± 0.95	20.17 ± 4.03	1.07 ± 0.21
	6	19.57 ± 0.75	13.25 ± 2.65	1.07 ± 0.21
	11	20.81 ± 2.6	8.77 ± 1.75	1.08 ± 0.22

Table S2. Experimental measurements for HIF-1α, STAT3 and AKT used in this study. Values are normalized with respect to measurements at time $t = 0$ h, corresponding to the steady states in untreated cells. STAT3 levels in (iv) were not detectable (n.d.) at $t = 11$ h and assumed to be zero.

[3]P. Kumar, U. Gullberg, I. Olsson, and R. Ajore. Myeloid translocation gene-16 co-repressor promotes degradation of Hypoxia-Inducible Factor 1. *PLOS One*, 10(5):e0123725, 2015.

[4]L. K. Nguyen, M. A. Cavadas, C. C. Scholz, S. F. Fitzpatrick, U. Bruning, E. P. Cummins, et al. A dynamic model of the Hypoxia-Inducible Factor 1α (HIF-1α) network. *J. Cell Scie.*, 126(6):1454–1463, 2013.

[5]A. Palazón, J. Aragonés, A. Morales-Kastresana, M. O. de Landázuri, and I. Melero. Molecular pathways: hypoxia response in immune cells fighting or promoting cancer. *Clin. Cancer Res.*, 18(5):1207–1213, 2012.

[6]G. L. Semenza. Regulation of physiological responses to continuous and intermittent hypoxia by hypoxia-inducible factor 1. *Exp. Physiol.*, 91(5):803–806, 2006.
